# The Emerging Roles of Steroid Hormone Receptors in Ductal Carcinoma in Situ (DCIS) of the Breast

**DOI:** 10.1007/s10911-018-9416-0

**Published:** 2018-10-18

**Authors:** Hugo Villanueva, Sandra Grimm, Sagar Dhamne, Kimal Rajapakshe, Adriana Visbal, Christel M. Davis, Erik A. Ehli, Sean M. Hartig, Cristian Coarfa, Dean P. Edwards

**Affiliations:** 1 Department of Molecular and Cellular Biology, Baylor College of Medicine, One Baylor Plaza, Houston, TX 77030 USA; 2 Department of Pathology and Immunology, Baylor College of Medicine, One Baylor Plaza, Houston, TX 77030 USA; 3 Avera Institute for Human Genetics, 3720 W 69th St, Sioux Falls, SD 57108 USA

**Keywords:** Steroid hormones, Estrogen receptor, Progesterone receptor, Ductal carcinoma In Situ

## Abstract

Ductal carcinoma in situ (DCIS) is a non-obligate precursor to most types of invasive breast cancer (IBC). Although it is estimated only one third of untreated patients with DCIS will progress to IBC, standard of care for treatment is surgery and radiation. This therapeutic approach combined with a lack of reliable biomarker panels to predict DCIS progression is a major clinical problem. DCIS shares the same molecular subtypes as IBC including estrogen receptor (ER) and progesterone receptor (PR) positive luminal subtypes, which encompass the majority (60–70%) of DCIS. Compared to the established roles of ER and PR in luminal IBC, much less is known about the roles and mechanism of action of estrogen (E2) and progesterone (P4) and their cognate receptors in the development and progression of DCIS. This is an underexplored area of research due in part to a paucity of suitable experimental models of ER+/PR + DCIS. This review summarizes information from clinical and observational studies on steroid hormones as breast cancer risk factors and ER and PR as biomarkers in DCIS. Lastly, we discuss emerging experimental models of ER+/PR+ DCIS.

## Introduction

**Fig. 1:**
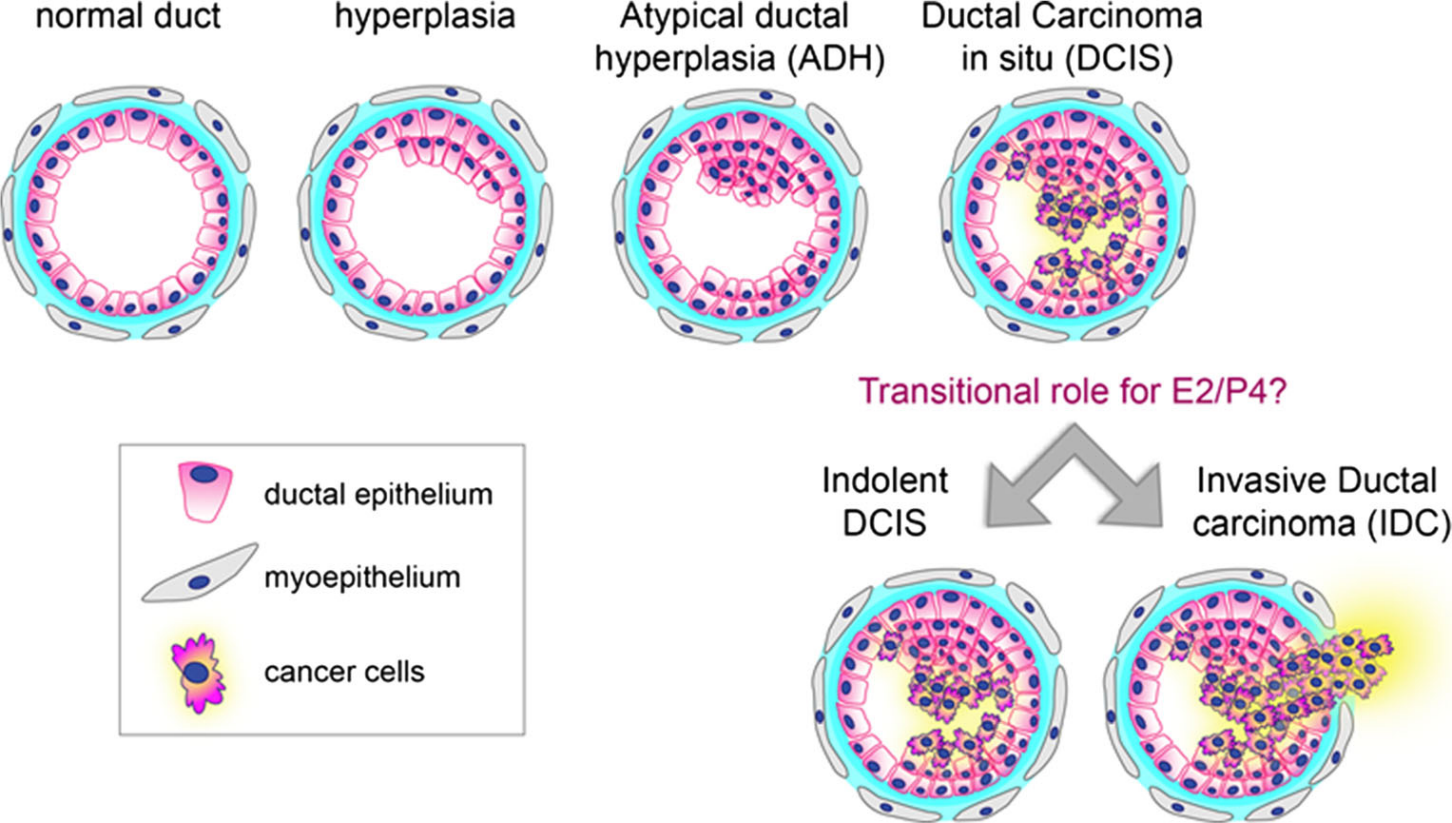
**Stages of breast cancer progression.** Simplified model of stages of breast cancer progression from normal ductal morphology, advancement to hyperplasia, non-obligate progression through atypical ductal hyperplasia, DCIS, and either arrest at in situ carcinoma or transition to IBC

Ductal carcinoma in situ (DCIS) is recognized as a non-obligate precursor to most types of invasive breast cancer (IBC) [1–6]. Intact DCIS is not inherently lethal and is defined by hyperproliferative cells confined to the lumen of mammary ducts. The Wellings model suggests these rapidly dividing cells most likely originate from normal luminal epithelial cells in terminal ductal lobular units (TDLUs) that acquire hyperplastic properties and progress to hyperplastic enlarged lobular units (HELUs) [7–9]. Hyperplasia is characterized as either usual or atypical, with the latter being more frequently associated with progression to invasive disease. Lee and colleagues in their study of alterations in gene expression of early hyperplastic precursors of breast cancer noted highly elevated expression of ERα in HELUs, which may be the fundamental defect responsible for widespread hyperplasia and the catalyst for further progression to more committed precursors of breast cancer [10]. Molecular studies have provided evidence that atypical ductal hyperplasia (ADH) is the earliest neoplastic multi-cellular benign lesion related to DCIS; and columnar cell lesions (CCLs) along with flat epithelial atypia and ADH are the missing links between normal breast tissue and DCIS in the low grade pathway [11–13]. In their study, Simpson et al. showed that CCLs share characteristics similar to some forms of low grade carcinoma (both in situ and invasive) representing a morphologic and molecular continuum between these lesions [14]. DCIS has distinct biologic and histologic characteristics that separate it from earlier precursors including varying degrees of cellular grading and these lesions are surrounded by a myoepithelial compartment that stains positive for markers such as p63 and smooth muscle actin [9, 15]. Pure DCIS can progress to the microinvasive stage involving myoepithelial cell breakdown along with a few cells breaching and traveling small distances past the myoepithelium and basement membrane (Fig. 1). Comparative genomic analysis of DCIS and IBC, including lesions from the same patient, show few differences in gene expression and DNA alterations indicating that the invasive potential of DCIS is largely pre-programmed genetically and that other unknown factors are responsible for transition of DCIS to invasive cancer [9, 16–23]. Development of more advanced screening technologies has resulted in a significant increase in the frequency of DCIS diagnosis over the past four decades. Whereas DCIS used to account for 1–2% of breast cancers in the early part of the twentieth century [9], DCIS now accounts for approximately 30% of all newly diagnosed breast cancers. Though it is estimated only one third of DCIS will progress to IBC without medical intervention [24], standard of care therapy is surgery and radiation, or adjuvant endocrine treatment. Histopathological assessments and molecular biomarkers have not been developed as yet that can reliably predict DCIS progression or recurrence [25]. Since DCIS represents a significant fraction of all newly diagnosed breast cancers, unnecessary treatment interventions impact a large number of women and remains a significant clinical problem.

DCIS shares molecular classifications with IBC including the estrogen receptor (ER) and progesterone receptor (PR) luminal subtype, which makes up approximately 70% of DCIS. Despite the established role and importance of ER and PR in luminal IBC, there is much less information on estrogen (E2) and progesterone (P4) and their cognate receptors in DCIS. Clinical trials demonstrate a significant effect of anti-estrogen therapy on recurrence prevention in women with ER+ DCIS [26]. Unlike IBC, the presence of PR added no predictive value [26], indicating divergent responsiveness of DCIS and IBC to steroid hormones. Furthermore, the molecular mechanisms underlying the transition of DCIS to IBC are not well defined and remain an underexplored area of research, partly due to the lack of suitable experimental model systems. In this review, we summarize clinical and observational studies on the roles of steroid hormones and ER/PR as biomarkers in DCIS. We also discuss the development and validation of steroid hormone responsive model systems of ER+/PR+ DCIS that mimic progression in humans.

### Steroid Hormones as Risk Factors for DCIS

The role of steroid hormones in the progression of DCIS is an understudied area, despite evidence suggesting they are potentially important drivers of breast disease in ER+ breast cancer [27–30]. The Women’s Health Initiative (WHI) randomized clinical trial, which assessed hormone replacement therapy (HRT) for breast cancer risk as a primary adverse outcome, included a large cohort of postmenopausal women with no prior hysterectomy [31]. This trial ended early due to findings that conjugated equine estrogen (CEE) plus medroxyprogesterone acetate (MPA) caused a significant increase in both coronary heart disease and IBC. Conversely, a parallel retrospective study found no increase in IBC from CEE treatment alone vs. placebo (5-year treatment regimen) in a large cohort of post-menopausal women with hysterectomy [32]. A follow-up retrospective analysis of the histological features and characteristics of breast cancers in the hormone-treated vs. placebo groups of the WHI trial suggest that CEE combined with MPA increased the number and size of invasive breast tumors [33]. This was attributed to an increase in cell proliferative cycles and breast epithelium density observed with prolonged use of CEE + MPA [34]. Few studies have assessed steroid hormone effects on the incidence of DCIS in postmenopausal women; however, a retrospective study of the WHI trial found a trend towards significance of higher incidence in a large cohort of women with no prior hysterectomy that were administered CEE and MPA compared to the placebo arm [33]. CEE alone had no effect on DCIS incidence compared to placebo in a mixed ER+/unknown PR status, or PR+/unknown ER status patient population [32]. While these clinical trial assessments indicate that progestins may be responsible for increased IBC (and possibly DCIS incidence), it must be noted that the patient cohorts in the CEE and MPA vs. CEE alone trials had intact and surgically excised uteri, respectively. Patients in the CEE alone trial were also mostly ER+, with less than half displaying PR+ status. While there was no data on how many patients displayed a positive score for ER and PR together, one can assume it was probably less than the number of patients displaying a PR+ status, which impacts the implication of results towards ER+/PR+ disease. In direct contrast to the negligible effects on proliferative disease in the CEE alone trial, laboratory in vivo studies showed that exogenous E2 supplementation stimulated proliferation of ER+ DCIS patient specimens xenografted in an athymic immune-deficient mouse model [35]. Furthermore, E2 increased proliferation of ER+ DCIS that are typically of the non-comedo, less aggressive type but had no effect on ER- DCIS specimens [35]. Collectively, these studies demonstrate the need for further investigation into the connections between steroid hormone signaling and DCIS risk.

Steroidogenic enzymes such as 17beta-hydroxysteroid dehydrogenase 1 (HSD17B1) and aromatase (CYP19A1) are frequently overexpressed in atypical ductal hyperplasia (ADH), DCIS, and IBC [36–41]. This provides a means for increasing intra-tumoral concentrations of E2 that may be a particularly important driver of ER+ DCIS and/or IBC in postmenopausal women [42]. Increased expression of aromatase in ADH and DCIS vs. normal breast tissues has been observed [41, 43], and a correlation was reported between aromatase overexpression and elevated in situ E2 concentrations in DCIS. These studies imply that similar to IBC, aromatization can generate a source of localized E2 capable of stimulating ER signaling pathways in DCIS of postmenopausal women.

Regulated expression of the aromatase gene is complex and can be controlled by a variety of different signaling molecules/pathways including IL-6, p38/JNK, GR, TNFα, JAK/STAT, and prostaglandin E_2_ (PGE_2_) [44–48]. These pathways are typically activated in adipose fibroblasts in the breast tumor microenvironment and are stimulated in response to signaling molecules from adjacent malignant epithelium as a mechanism to sustain aromatase expression and local E2 synthesis. However, the prostaglandin E_2_-synthesizing enzyme cyclooxygenase 2 (COX-2) is expressed in DCIS and IBC and has been observed to be associated with progression and recurrence and with up-regulation of aromatase [45, 48–51]. Overexpression of COX-2 in ER + MCF-7 breast cancer cells significantly increased the expression of aromatase, an effect blocked by a COX-2 inhibitor [51]. A role for COX-2 in breast cancer has been demonstrated using ER+ and ER- breast cancer cell lines in which the COX-2 inhibitor Celecoxib was found to increase apoptosis and to reduce tumor growth [49]. Furthermore, studies of transgenic mice overexpressing COX-2 in the mammary gland have shown that COX-2 is sufficient for induction of mammary tumorigenesis [52, 53]. Clinical trials have reported the co-targeting of COX-2 and aromatase in a neo-adjuvant setting in ER+ DCIS patients. No clinical benefit of Celecoxib, either alone or in combination with the aromatase inhibitor (AI) Exemestane, was noted in a randomized trial when agents were given over a short course of 14 days prior to surgery [54]. However, in another study, the combination of Celecoxib and Exemestane administered over 12 weeks was more effective at reducing proliferation of ER+ DCIS compared to Exemestane alone [55]. Of note, the first study cohort included cases of pure DCIS plus some DCIS lesions with associated invasion, while the second study comprised cases with pure DCIS only. Both trials were adequately powered to observe statistical differences, negating any discrepancies due to patient numbers in each cohort. These data suggest co-targeting COX-2 and aromatase may have a benefit with long-term treatment regimens in patients with pure ER+ DCIS. Interestingly, COX-2 upregulation has also been described in stem-like cell spheroid cultures derived from patient DCIS lesions (including ER+/PR+ DCIS) and mechanistic studies suggested that COX-2 promoted the enrichment of stem-like cells [56, 57]. While an association between a hormonal response and COX-2 expression and activity has been known for a few years (i.e. mammary glands of pregnant and E2 plus P4 treated rats, DMBA-induced rat mammary tumor growth, and in ER+ breast cancer cells) [58–60], the recent clinical data collectively suggest COX-2 may be an important driver of ER+ breast cancer progression.

### Steroid Hormone Receptors and Aromatase as Biomarkers and Therapeutic Targets in DCIS

Similar to IBC, DCIS lesions are routinely analyzed for the expression of ER and PR and approximately 70% are ER+/PR+. Furthermore, clinical studies have determined that ER/PR levels in DCIS significantly correlate with tumor grade (i.e. high ER+/PR+ is associated with a lower grade and vice-versa) [61]. Clinical studies have also observed that ER and PR are upregulated in HELUs (early lesion precursors) and that Ki67 is significantly increased compared to normal terminal ductal lobular units (TDLU). These observations suggest that steroid hormones may be responsible for hyperplastic growth of normal breast epithelial cells that may ultimately progress through atypical ductal hyperplasia and DCIS [62]. Moreover, smaller DCIS lesions tend to be ER+ and to have a non-comedo histopathology associated with a less aggressive phenotype [61, 63]. ER+/PR+ positivity in both DCIS and IBC also correlates with favorable prognosis because these tumors follow a low grade trajectory and tend to exhibit well-differentiated morphology even with a high nuclear grade [61, 63, 64].

The predictive value of ER and PR expression in breast cancer has been appreciated for many years and serves to inform clinicians whether a patient is a candidate for endocrine therapy [65]. High ER expression in epithelial cells of hormone receptor positive breast cancer patient samples indicates cancer cells are dependent on E2 for continued growth, and targeting these cells with antiestrogens such as Tamoxifen or AIs such as Letrozole or Anastrozole will likely shrink tumors and improve patient outcome. Tamoxifen use in postmenopausal women presenting with early-stage ER+ breast cancer is an effective therapy in many patients [66]. Meta-analysis of clinical trials where AI was given either 2–3 years after Tamoxifen or in lieu of Tamoxifen for five years, show a greater reduction in recurrence of ER positive breast cancer than Tamoxifen alone [66]. Similar to invasive breast cancer, ER+/PR+ positive DCIS with this diagnosis have been treated with anti-hormonal therapy and demonstrated to have reduced recurrence of subsequent breast cancer, indicating DCIS lesions in these patients are hormone responsive [26].

Clinical trials such as the NSABP-B24 have shown a significant reduction of subsequent breast cancer recurrence in a cohort of women with ER+ DCIS treated with Tamoxifen [25, 26, 67], whereas patients with ER-DCIS derived no benefit. In the NCICCTG MAP.3 prevention trial that included patients diagnosed with DCIS, the efficacy of the AI, Exemestane, was assessed for prevention of ER+ invasive breast cancer [68]. Results reported fewer invasive breast cancers in the Exemestane cohort compared to placebo. Further double blind prospective trials have compared the outcomes of Tamoxifen vs. AIs like Anastrazole. The NSABP B-35 trial comparing Tamoxifen to Anastrozole in an adjuvant setting for treatment of ER+ DCIS. Patients that had undergone lumpectomy followed by radiation observed that Anastrozole had a benefit over Tamoxifen in improving breast cancer-free interval in postmenopausal patients that were younger than 60 years of age [69]. The IBIS-II trial, also comparing adjuvant Tamoxifen to Anastrazole, did not find a significant difference in breast cancer recurrences between the two cohorts, but suggested that Anastrozole resulted in fewer ER+ invasive tumor recurrences compared to Tamoxifen [70]. Both trials noted fewer breast cancer recurrent events indicating Anastrozole might be a better adjuvant option for ER+ DCIS patients, especially in younger, postmenopausal women. While the biological explanation for this advantage of Anastrozole over Tamoxifen is unknown, it merits follow-up, mechanistic studies to interrogate aromatase in this patient population to determine whether aromatization and local E2 production at disease sites can be leveraged to more effectively treat these patients. Randomized trials in postmenopausal women that received adjuvant Tamoxifen post-operatively for five years observed a longer disease-free survival after receiving sequential Letrozole for an additional 2.4 years [71]. These trials suggest there is a benefit to blocking E2 signaling in postmenopausal women with ER+ DCIS. Furthermore, clinical studies identified COX-2 as a potential marker of early relapse and AI resistance in patients with ER+ DCIS, suggesting the potential value of co-targeting COX-2 and aromatase as a therapeutic strategy [55].

PR expression in IBC is well established as an independent prognostic marker of better survival and disease-free interval. As an E2 regulated target gene, PR adds predictive value to ER as a marker of responsiveness to endocrine therapy. There is some question however as to whether the presence of PR in ER+ DCIS patients adds predictive value to Tamoxifen treatment [26] as it does in IBC [72]. PR also mediates independent actions of progesterone in breast cancer and responses are highly variable dependent upon the context. Clinical studies performed decades ago showed that high doses of synthetic progestin agonists were as effective as Tamoxifen for second line treatment of advanced breast cancer [73, 74]. However, since synthetic progestins in combination with estrogens in HRT increased breast cancer incidence [32–34], their use in an adjuvant setting for treatment of early stage breast cancer has been discouraged. The use of PR antagonists for adjuvant treatment of primary breast cancer has been complicated by substantial side effects that resulted in termination of clinical trials so their potential efficacy remains unknown. Newer generation PR antagonists have been developed with lower anti-glucocorticoid receptor activities that may be of promise [75]. Progesterone can also act to antagonize the growth promoting effects of E2 in breast cancer through cross-talk between PR and ER. Studies have shown a physical interaction between PR and ER by co-immunoprecipitation and other pull down assays as a mechanism by which PR attenuates ER action [76–78]. Genomic studies have shown that cross-talk between PR and ER in breast cancer cell lines and primary cells derived from patient breast tumors involves an extensive redirecting by PR of where ER binds on chromatin sites resulting in a reprogramming of target gene expression that attenuates the proliferative and growth promoting actions of E2 [79, 80]. More recent studies with ER+/PR+ patient derived xenograft mouse models showed that chronic treatment with progesterone or synthetic progestins in vivo attenuated the growth stimulatory actions of ER also by a redistribution of ER and PR chromatin binding sites and blunting of ER-mediated target genes [81]. In addition, PR was discovered to associate with RNA polymerase III and to decrease tRNA transcribed target genes in response to progestin as an additional indirect mechanism by which PR impedes the action of ER [81]. These apparent conflicting roles of progesterone and PR may reflect different responses in early and late stage of breast cancer progression and has complicated the use of PR as a therapeutic target [82, 83]. However, co-targeting ER and PR with respective antagonists has been implicated from PR-ER cross-talk studies. While the value of PR as a biomarker and potential therapeutic target in invasive breast cancer is well-appreciated [82–85], there is little information on a role for progesterone and PR in the development or progression of DCIS. Thus, continued investigation on the role of hormone receptor biology in DCIS may greatly benefit patients with this disease.

### ER/PR Positive Experimental Animal Models of Progression of Pre-Invasive Breast Disease to Invasive BC

The development of hormone receptor positive breast cancer models in mice has been a challenge due to the loss of ER/PR in most genetically engineered mouse models (GEM). It is even more challenging to find suitable models that express ER and PR and progress through the early stages of breast cancer in a manner that mimics human disease. Mice with a germline deletion of p53 typically die early from thymic lymphomas before mammary tumors develop [86]. However, mammary gland tissue from p53-null mice transplanted into the cleared fat pad of syngeneic wild-type Balb/C mice allows long-term studies of mammary tumor progression. Using this transplant method, spontaneous adenocarcinomas of ductal origin arise from the loss of p53 and progress through ductal hyperplasia and DCIS before becoming invasive breast cancer. The tumors retain expression of ER and PR, and are frequently aneuploid [87]. Interestingly, P4 treatment but not E2 treatment led to increased aneuploidy [88]. This model has been instrumental for establishing how ovarian hormone signaling affects development of hormone receptor-positive tumors. In contrast to wild-type BALB/c, p53-null mammary epithelium is highly susceptible to tumor induction by E2 and P4, either alone or in combination [89]. Serial transplantation of these p53-null hyperplastic outgrowths led to the development of pre-neoplastic outgrowth lines, designated PN, which have varying degrees of tumorigenic potential [90]. While this model has been useful in studying early breast disease, it has not been characterized as a suitable model to study DCIS transition to IBC due to the weak tumorigenicity and low tumor incidence.

Other models of spontaneous ER/PR positive mammary tumor development that progress through DCIS include chemical carcinogen induced tumors in rodents and transgenic mice. The use of chemicals to induce ER+/PR+ mammary tumors offers the advantage of generating tumors that are not only responsive to hormones, but behave much like human tumors by generating a variety of histopathologies reminiscent of human DCIS [91, 92]. However, chemical carcinogenic models have not been used specifically to study ER+/PR+ DCIS and transition to IBC. While these models are attractive, they require use of rats, which are cumbersome and until recently, were challenging to manipulate genetically. Overexpression of the AIB1 (SRC-3) oncogene in mice under the control of MMTV promotes the formation of ER+ ADH and DCIS-like lesions. While these lesions retain ER in the absence of E2, they do not progress to invasive disease suggesting that they are dependent on E2 to achieve a transition from in situ to invasive disease [93, 94]. The SRC-3 mouse model has low PR expression that is insensitive to stimulation by E2, thus limiting its use to modeling ER+/PR- DCIS [93]. A GEM model that spontaneously develops ER+/PR+ tumors as a result of targeted disruption of the STAT1 gene has been reported [95]. Subsequent characterization of mammary tumor histopathological features demonstrated this mouse model gives rise to mammary intraneoplasias that progress to carcinomas in a manner similar to the transition of DCIS to IBC in humans [96]. Tumor cells derived from STAT1^−/−^ mice retain ER and PR expression and E2 is required for successful engraftment and progression of subsequent tumor transplants that resemble luminal-like human breast cancers [96]. Responsiveness of STAT1^−/−^ tumors to progesterone was not reported. More recently, a transgenic mouse line was developed that conditionally expresses an activating mutation in Ki-Ras^G12V^ in mammary epithelium after lactation by use of the beta lactoglobulin promoter. These mice develop invasive ductal adenocarcinomas with a high frequency at 3–9 months after lactation and the tumors exhibit luminal A subtype characteristics including high levels of ER and PR. Cell lines derived from these tumors retain ER and PR and are responsive to E2 in vitro and grow in nude mice as tumor xenografts that are inhibited by the anti-estrogen ICI 182780. This model system however has not been evaluated as yet for whether tumors progress through DCIS or for responsiveness to progesterone [97].

Although tumor xenografts implanted in cleared mammary fat pads of immunocompromised mice with ER+/PR+ metastatic breast cancer cell lines, or patient-derived tumor specimens, have been used extensively to examine the role of ER and PR in progression of invasive breast cancer, this is not optimal as a DCIS xenograft model since the natural ductal microenvironment has been removed. The need for a more suitable DCIS xenograft model to study the progression of human DCIS to invasive cancer in vivo has been addressed by Behbod et al., who introduced the mouse mammary intraductal DCIS (MIND) xenograft, that mimics the progression of human DCIS within the natural microenvironment of the mammary ductal epithelium [98–100]. Human DCIS epithelial cells from patients or cell lines are injected into the primary duct of immunocompromised mice and engrafted cells readily form DCIS lesions with histopathological characteristics similar to human disease. Xenografts must survive within the hypoxic and nutrient-deficient microenvironment of the mammary gland duct and invade into the stroma by breakdown of the surrounding myoepithelial cell layer and the basement membrane. The MIND system differs significantly from more traditional mouse PDX models that implant breast cancer cells or tumors into the mammary fat pad cleared of epithelium and thus are inadequate models to explore the progression of pre-invasive lesions since the natural barriers to overcome invasion have been removed. The MIND system has also been used to analyze the effects of various genes on transition of pure DCIS to invasive tumors in vivo [100, 101]; however, it has only been used in a limited manner up until now to examine the role of steroid hormones on transition of DCIS to invasive cancer due to the previous lack of an ER+/PR+ human DCIS cell line (see below). The MIND method has been used to xenograft metastatic ER+/PR+ breast cancer cell lines in a more “native” ductal microenvironment [102]. Although DCIS-like lesions were formed with these cell lines that eventually progressed to invasive and metastatic foci, this model system for study of DCIS is inherently limited by an “invasive ductal carcinoma program” since the cells injected intraductally were derived from invasive breast cancers. An additional xenograft model that reported progression through DCIS includes human mammary epithelial cells obtained from reduction mammoplasties that were transformed by expression of an oncogene and then intraductally injected into mammary glands of immunodeficient mice to form DCIS lesions. While these lesions were positive for ER, they did not express PR which limited studies to ER+/PR- DCIS [103].

## ER/PR Positive Models of Human DCIS

**Fig. 2:**
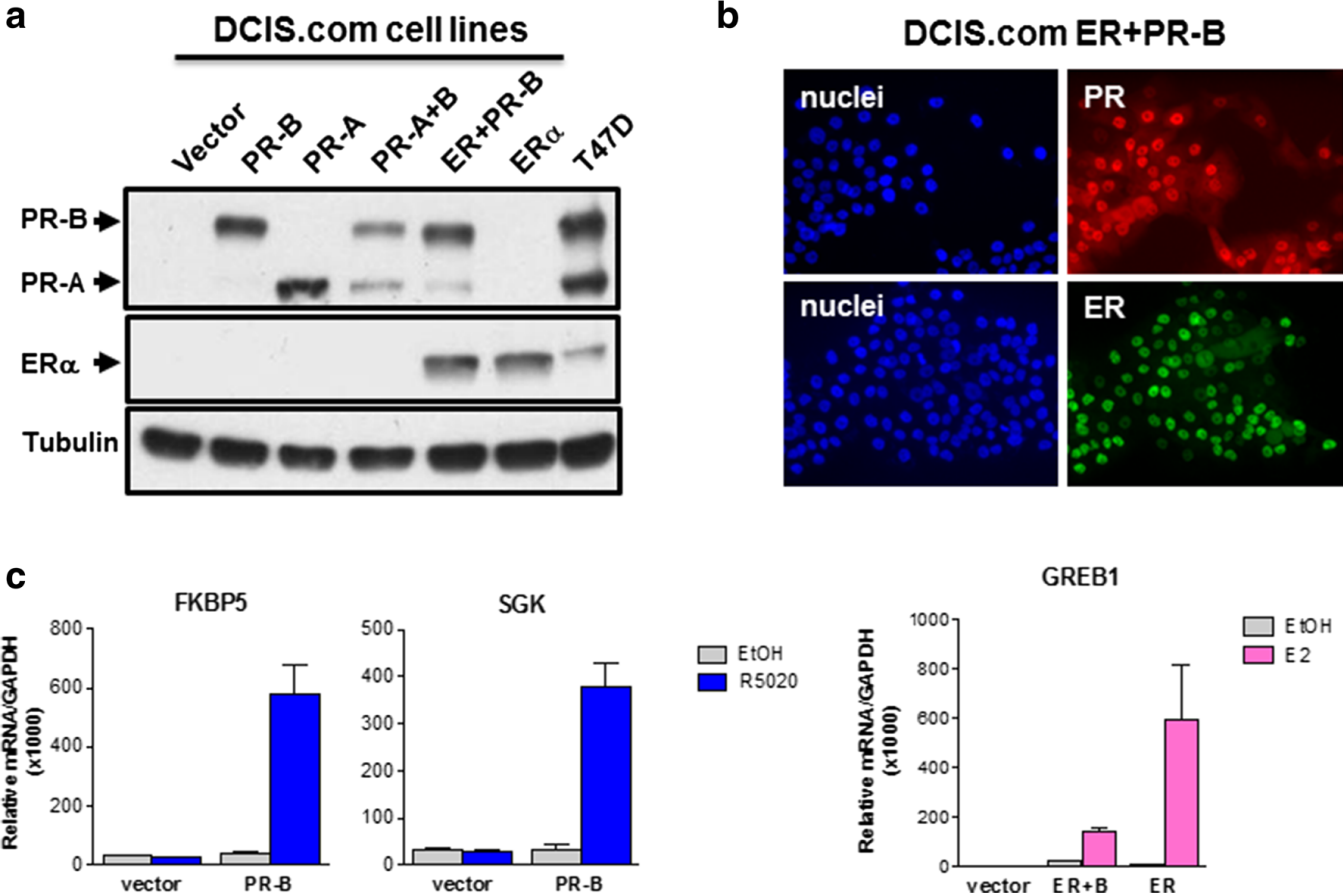
**ER and PR expression and R5020 response in engineered human DCIS.COM cells.** Lentivirus transduction and cell sorting was used to stably express different combinations of ERα/PR including PR (A or B isoforms), ERα alone or both ERα and PR in DCIS.COM cells. STR DNA fingerprinting was done by the CCSG-funded Characterized Cell Line Core at M.D. Anderson Cancer Center (NCI # CA016672) to validate the cell lines as breast cancer epithelial cell origin. Expression of PR or ER is shown by immunoblot analysis in panel (**a**). Immunofluorescent labeling of ER+/PR+ DCIS.COM cells demonstrates that ER and PR are each expressed in nuclei of the majority of cells, Scale bar: 50 μm (**b**). These engineered cell lines are responsive to the synthetic progestin R5020 or 17β estradiol (E2) in terms of induction of known target gene expression by qRT-PCR after 24-h hormone treatment (**c**)

A limitation in understanding the role of E2 and P4 on transition of DCIS to IBC is the lack of ER+/PR+ human DCIS cells lines for molecular mechanism studies and for use in the MIND xenograft system. Of the few human DCIS cell lines available, none express ER and PR including the well-characterized DCIS.COM (comedo) cell line [104]. In our laboratory, we stably expressed different combinations of human ER/PR including PR (A or B isoforms), ERα, or both ERα and PR in DCIS.COM cells. The parent cells were transduced with a lentiviral vector expressing PR or ERα, driven by the E1F-α promoter, which included a bright fluorescent protein (ZsGreen or Tomato Red) expressed from an IRES, as described by Welm *et al.* [105]. Transduced cells were FACS-sorted for the fluorescent protein, and were confirmed to express intact PR or ERα in the majority of sorted cells. Immunoblot assays in the different engineered cell lines verified ER/PR expression levels that were similar to endogenous receptors in T47D breast cancer cells and lack of receptors in parental and vector control DCIS.COM cells (Fig. 2a). As shown by immunofluorescence of cells grown on coverslips, ER and PR were both expressed predominantly in the nuclei as anticipated (Fig. 2b).

**Fig. 3:**
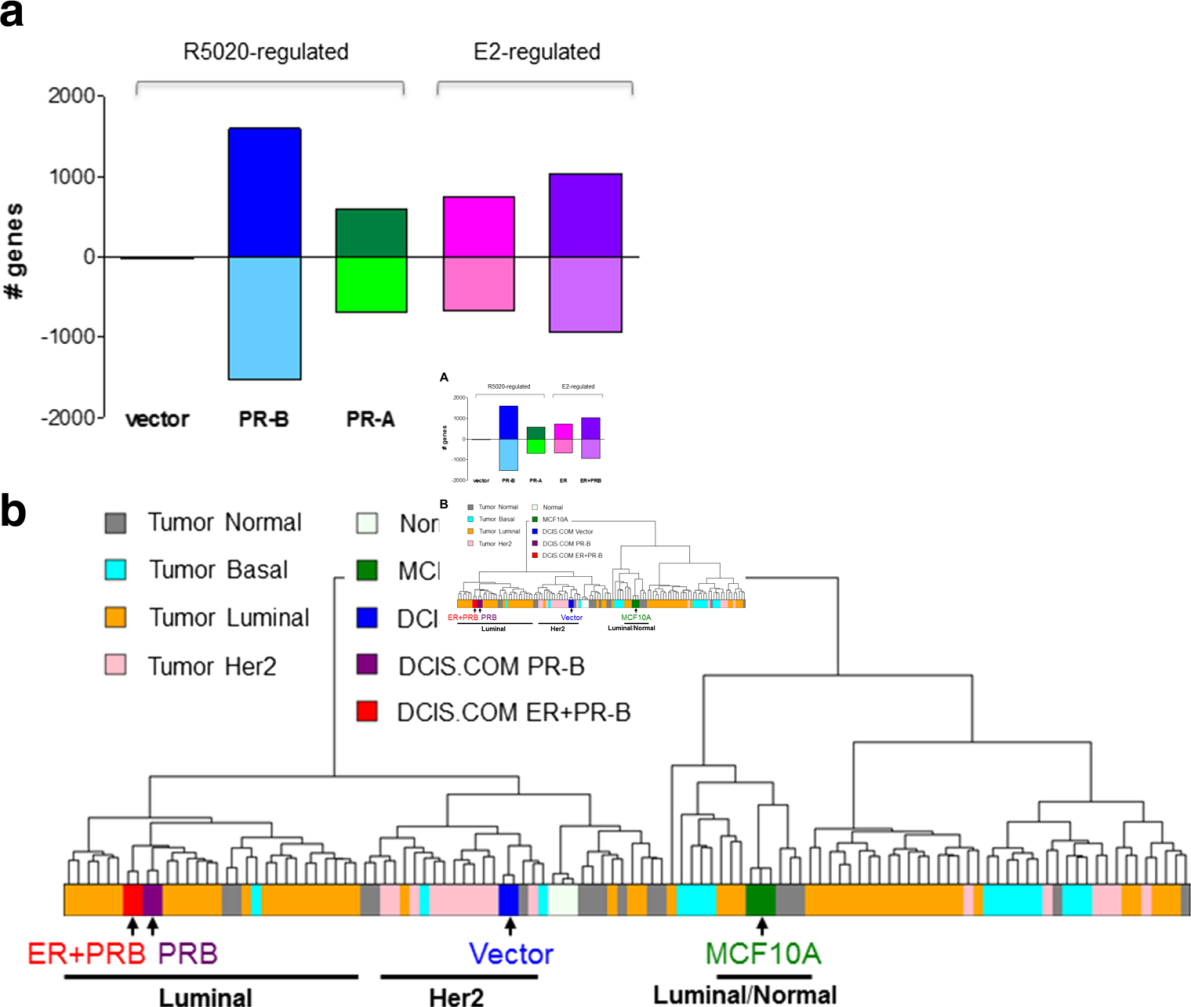
**Global gene expression analysis in engineered DCIS.COM cells. a** Summary of gene expression changes found by microarray analysis of the DCIS.COM cell lines after a 24-h hormone treatment. The Illumina HumanHT-12 v4.0 Gene Expression Beachchip Assay was used. Genes were selected based on the criteria of a fold-change greater than 1.25 with a *p* value of less than 0.05. The patterned areas indicate commonly regulated genes, with the solid color showing genes uniquely expressed in that cell line. **b** Dendrogram integrating our gene expression profiling of ER+/PR+ DCIS.COM cells with a public specimen cohort of patient DCIS and tumor samples (normal-like, basal, HER2-enriched, and luminal subtypes)

PR positive DCIS.COM cells are highly responsive to the synthetic progestin R5020 (and natural P4) as demonstrated by induced expression of known PR target genes, including as examples *FKBP5* and *SGK* (Fig. 2c). ER+/PR+ DCIS.COM cells are also responsive to E2 as indicated by upregulation of a known ERα target gene such as *GREB1* (Fig. 2c). Microarray gene expression profiling was conducted to explore global gene expression changes in response to treatment with steroid hormones. In the DCIS.COM PR-B+ cell line, R5020 stimulated a robust set of unique genes compared to the PR-A cell line (Fig. 3a). In cells engineered to express ER alone, or both ER and PR-B, E2 stimulated robust gene expression changes in both cell lines (Fig. 3b). The microarray data was also used to determine the molecular signature of PR-B+ and ER+/PR-B+ DCIS.COM cell lines as compared with parental cells and invasive breast cancer specimens from the Cancer Genome Atlas (TCGA) database (Fig. 3b). Parental DCIS.COM cells have a molecular signature reminiscent of basal/HER2 subtype rather than a luminal subtype. As shown by the dendrogram in Fig. 3b, ER+/PR-B+ and PR-B+ DCIS.COM cells shift away from the basal/HER2 molecular signature and cluster with luminal breast (A and B) cancer. Our engineered ER+/PR-B+ cells lines have decreased expression of basal markers such as keratin 5 and 14, and induce expression of the luminal marker mucin 1. Other markers for luminal cells (EpCam, keratin 19) and basal cells (keratin 17, p63) are unchanged. R5020 treatment of PR-B+ cells negatively correlated with an EMT gene signature, whereas E2 treatment of the ER + PR-B+ cells did not. T47D cells treated with P4 and E2, as compared to E2 treatment alone, also negatively correlated with an EMT gene signature [79].

The ER+/PR+ DCIS.COM cell line has been used in the MIND system and is responsive to hormones in vivo. Combined E2 and P4 treatment of DCIS xenografts formed by intraductal injection of ER+/PR+ DCIS.COM cells stimulated up-regulation of a NEMO/NF-κB/IL-6 pro-inflammatory pathway that relied on NEMO to maintain expression of the PML tumor suppressor. Knock-down of NEMO in ER+/PR+ DCIS.COM cells prior to intraductal xenografting increased invasive progression of DCIS lesions *in vivo*, implicating NEMO as a potential tumor suppressor regulated by E2 and P4 in the transition of DCIS to IBC [100]. These data collectively validate the engineered ER+/PR+ DCIS.COM cell lines as a physiologically relevant model. The MIND system has also been used successfully for intraductal engraftment of primary ER+/PR+ DCIS epithelial cells derived from patients. The xenografts retain expression of receptors after implantation [99] and exhibit a full spectrum of human DCIS histopathologies similar to that of the original patient DCIS specimens [99]. Importantly, a fraction of the xenografts showed invasive progression in mice which provides the opportunity to examine the influence of hormones and hormone antagonists on the invasive potential of patient derived ER+/PR+ DCIS [98, 99].

## Conclusion

Increased access to breast mammograms world-wide is responsible for the higher incidence of detection of DCIS that currently accounts for approximately one third of all breast cancer cases [106, 107]. The rise in incidence highlights the importance of better understanding DCIS pathology, including the factors that promote transition to IBC. Standard clinical management of DCIS patients includes surgical removal of lesions, radiation therapy, and in some cases, endocrine therapy for ER+ DCIS. These options are somewhat rudimentary in the age of personalized medicine considering that the majority of women with DCIS will not experience invasive cancer if left untreated. Thus, over diagnosis and over treatment of women with DCIS is a chief clinical challenge. Efforts to define the critical molecular factors that regulate the transition from DCIS to invasive disease have been hindered by lack of suitable experimental systems that recapitulate this process in either in vitro or in vivo with animal models. Several questions remain unanswered regarding DCIS biology including the role that steroid hormones and their cognate receptors play in the development and/or progression of in situ carcinoma. A potentially important difference in steroid hormone biology between pre- and post-menopausal women with DCIS is that women are exposed to combinations of E2 and P4 for many years until menopause, at which point E2 responses in DCIS may frequently switch to in situ rather than endocrine sources. Data from clinical trials have established the diagnostic and therapeutic value of ER expression in DCIS patients [26], while the potential role of PR remains largely unknown. Various experimental models of ER+/PR+ breast cancer progression have been described in the literature and some are highlighted in this review, but most are not ideal for studying the role and mechanisms of steroid hormones and their cognate receptors in the transition of DCIS to IBC. The ER+/PR+ human DCIS cell lines described here together with the mouse intraductal DCIS (MIND) xenograft model provide opportunities to advance studies and our understanding of how steroid hormone signaling pathways may be useful to identify biomarkers to better stratify DCIS patients for treatment options and for development of strategies for prevention of DCIS progression to invasive breast cancer.

## Abbreviations

IBC: Invasive breast cancer
DCIS: Ductal carcinoma In Situ
ER: Estrogen receptor
PR: Progesterone receptor
E2: 17β-estradiol
P4: Progesterone
HRT: Hormone replacement therapy
GEM: Genetically engineered mice
MIND: Mouse mammary intraductal DCIS xenograft
